# Staging Alzheimer's disease using the Functional Assessment Screening Tool (FAST): a crosswalk with the Montreal Cognitive Assessment (MoCA)

**DOI:** 10.1002/alz70857_107045

**Published:** 2025-12-25

**Authors:** Brant Mittler, Ying Wang, Joel Reisman, Dan Berlowitz, Peter J Morin, Raymond Zhang, Amir Abbas Tahami Monfared, Quanwu Zhang, Weiming Xia

**Affiliations:** ^1^ Geriatric Research Education & Clinical Center, VA South Texas Healthcare System, San Antonio, TX, USA; ^2^ Wentworth Institute of Technology, Boston, MA, USA; ^3^ University of Massachusetts Lowell, Lowell, MA, USA; ^4^ Boston University Chobanian & Avedisian School of Medicine, Boston, MA, USA; ^5^ Clinical Evidence Generation, Deep Human Biology Learning, Eisai Inc., Nutley, NJ, USA

## Abstract

**Background:**

The Functional Assessment Screening Tool (FAST) evaluates functional changes over the course of Alzheimer's disease. This study aimed to evaluate the extent to which FAST scores correspond to cognitive disease staging by the Montreal Cognitive Assessment (MoCA) test.

**Method:**

Paired MoCA and FAST scores were analyzed in patients with mild cognitive impairment (MCI) or Alzheimer's dementia (AD) in the Veteran's Affairs Healthcare System (2020–2024). FAST score stage cut‐off means, standard deviations (SD), medians, and ranges were generated. Linear and repeated measures mixed effects analysis was performed adjusting for patient demographics.

**Result:**

The study sample (*N* = 405) had a mean age of 77.9 years (95.6% men, 11.1% Black, and 6.2% Hispanic). MoCA cut‐offs for Normal, MCI, Mild, Moderate, and Severe AD stages were ≥29, 26,18, 11 and ≤10, respectively. Corresponding mean (SD) and median FAST score cut‐offs separating disease stages were: normal (*n* = 1), 2.0 and 2; MCI (*n* = 21), 3.3 (1.7) and 3; mild (*n* = 206), 3.8 (1.5) and 4; moderate (*n* = 118), 4.8 (1.4) and 5; severe (*n* = 59), 5.8 (1.0) and 6. FAST tests resulted in score ranges of 1–7 for MCI–moderate stages and 4–7 for severe AD. Of note, more than half the patients (*n* = 205) scored in the normal/preclinical ranges of FAST (1 or 2); while, only 1 patient scored normal on the MoCA. FAST linear least squares (LS) means were distributed as <3.6, 3.6‐<4.0, 4.0‐<5.1, 5.1‐6.0, >6 for normal, MCI, mild, moderate, and severe stages projected by MoCA cut‐offs, respectively (adjusted R‐squared=0.25). The LS means from categorical regression were 2.8, 4.2, 4.5, 5.5, and 6.5, normal, MCI, mild, moderate, and severe stages, respectively (adjusted R‐squared=0.24). Similarly, LS means from repeated measures analysis found FAST score ranges for normal to severe AD were <3.9, 3.9‐<4.2, 4.2‐<5.1, 5.1‐5.9, and >5.9.

**Conclusion:**

This study identified FAST score thresholds corresponding to clinical stages from normal to severe AD according to a regressional crosswalk with MoCA. FAST provides a global functional assessment; however, our data suggest that it may underestimate disease severity relative to MoCA. Nonetheless, FAST is widely used in clinical practice, providing an alternative clinical tool for staging disease progression.

